# Prevalence and factors associated with depressive and anxiety symptoms among Palestinian medical students

**DOI:** 10.1186/s12888-020-02658-1

**Published:** 2020-05-19

**Authors:** Ramzi Shawahna, Suhaib Hattab, Rami Al-Shafei, Mahmoud Tab’ouni

**Affiliations:** 1 Department of Physiology, Pharmacology and Toxicology, Faculty of Medicine and Health Sciences, An-Najah National University, New Campus, Building 19, P.O. Box 7, Nablus, Palestine; 2 An-Najah BioSciences Unit, Centre for Poisons Control, Chemical and Biological Analyses, An-Najah National University, Nablus, Palestine; 3 Department of Medicine, Faculty of Medicine and Health Sciences, An-Najah National University, Nablus, Palestine

**Keywords:** Anxiety, Depression, Medical students, Palestine, Middle East

## Abstract

**Background:**

Co-existence of depression and anxiety can be associated with severe detrimental consequences to the physical, mental and social wellbeing of the affected populations. This study was conducted to determine prevalence of depressive and anxiety symptoms among Palestinian medical students and to investigate associations between sociodemographic factors of the students with depressive and anxiety symptoms.

**Methods:**

This study was conducted in a cross-sectional observational design using a questionnaire in the period between September 2018 and April 2019 in a major university in the West Bank of Palestine. Depressive symptoms were assessed using the Beck Depression Inventory-II (BDI-II) and anxiety symptoms were assessed using the Beck Anxiety Inventory (BAI). The questionnaire also collected the sociodemographic characteristics of the students. Reliability of the questionnaire was tested using the test re-test method. A total of 425 medical students were invited to participate in the study.

**Results:**

Of those invited, 286 students completed the questionnaire, giving a response rate of 67.3%. More than half (56.6%) of the students had minimal depression, 20.3% had mild depression, 14.0% had moderate depression, 9.1% had severe depression, 23.4% had no anxiety, 29.7% had mild to moderate anxiety, 25.5% had moderate to severe anxiety, and 21.3% had severe anxiety. Multiple linear regression analysis showed that academic stage (*p*-value < 0.01), Grade Point Average (*p*-value < 0.01), mental health status (*p*-value < 0.001), ever attempted suicide (*p*-value < 0.05), and religious commitment (*p*-value < 0.01) were predictors of BDI-II scores. Multiple linear regression analysis showed that academic stage (*p*-value < 0.05) and mental health status (*p*-value < 0.001) were predictors of BAI scores.

**Conclusions:**

Depressive and anxiety symptoms were prevalent among Palestinian medical students in a major university in the West Bank of Palestine. Interventions might be designed to improve self-rated mental health of medical students in their academic years, ameliorate study conditions, and provision of counseling services to improve spirituality might be effective in reducing symptoms of depression and anxiety among medical students in Palestine. Future studies are still needed to investigate if these interventions could be useful in reducing depressive and anxiety symptoms among Palestinian medical students.

## Background

Psychological distress among university students has been a concern for public health authorities around the globe [1]. Among university students, medical students are particularly more vulnerable to psychological distress and morbidity [1–7]. Previous studies have shown that depression and anxiety are highly prevalent among medical students in different countries around the world [2–25] including countries in the Middle East [2, 3, 16, 21–25].

A recent meta-analysis of 77 different studies reported a global depression prevalence rate of 28.0% among medical students [18]. Some studies have reported alarming rates of depression and anxiety among medical students. A study conducted at the Royal College of Surgeons in Bahrain showed that 40% of participants had depressive symptoms and 51% had anxiety symptoms [2]. Similarly, a study conducted at the Foundation University Medical College in Pakistan reported that 51% of students had depression and anxiety [4]. Other studies have reported prevalence rates of depression and anxiety above 35% among medical students [1, 6, 15].

Prevalence rates of depression and anxiety were shown to be higher among medical students compared to their age-matched peers in both general population and those studying non-medical subjects [6, 7, 26–28]. Such high prevalence rates of depression and anxiety among medical students were attributed to personal and/or institutional factors [29]. Depression is characterized by many symptoms like sadness, excessive loss of interests and/or pleasure, feeling guilty or low self-esteem, disturbances in sleep and/or appetite, feeling tired, and decreased ability to concentrate [30, 31]. Anxiety is characterized by an inappropriately excessive and/or persistent worry that is often accompanied by symptoms like tachycardia, tremors, restlessness, fatigue, poor concentration, irritability, and disturbances in sleep pattern [32]. Co-existence of depression and anxiety can be highly disabling and might result in severe detrimental consequences to the physical, mental and social wellbeing of the affected populations [33, 34]. Depression and anxiety might have severe adverse consequences on the academic performance of medical students, their reported quality of life, contribute to substance abuse, decrease empathy, and academic dishonesty [1, 35, 36]. Previous studies have also shown that poor mental health was a predictor of later psychological distress among practicing physicians [37, 38]. Considering the severity of these detrimental consequences, understanding the prevalence of depression and anxiety among medical students in Palestine becomes imperative before designing and implementing interventions to improve mental health, create supportive environments, and reduce psychological distress among medical students.

While there were many studies investigating the prevalence of depressive and anxiety symptoms among medical students and the potential sociodemographic characteristics associated with high prevalence rates in different countries including some Middle Eastern countries, little is known on the prevalence of depression and anxiety among Palestinian medical students. Moreover, little is known on the sociodemographic factors that could be associated with depressive and anxiety symptoms among Palestinian medical students. Therefore, this study was conducted to determine the prevalence of depressive and anxiety symptoms among Palestinian medical students and to investigate associations between sociodemographic factors and depressive and anxiety symptoms.

## Methods

### Study context

The study was conducted in one of the major universities in the West Bank of Palestine. The Doctor of Medicine (MD) program at the university where the study was conducted is a 165-credit hour program that students often complete in 6 academic years. Students complete 135 credit hours of basic courses and 130 credit hours of clinical courses and training. Graduates of the MD program are licensed to assume practitioner roles in all healthcare establishments providing primary, secondary, and tertiary healthcare services after completing a compulsory hospital-based training program. The present study was conducted in the period between September 2018 and April 2019 to assess prevalence of depressive and anxiety symptoms among the medical students. The study period covered both 1st and 2nd semesters of the academic year 2018/2019. All students enrolled in the MD program at the time of the study were either Palestinians who were born and raised in the West Bank of Palestine or had Palestinian heritage. Students identified themselves as Muslims or Christians.

### Study settings

Students in the basic stage (academic year 1 to academic year 3) of medical school were recruited on campus. Students in the clinical stage (academic year 4 to academic year 6) were recruited in their training sites in different hospitals in the West Bank of Palestine.

### Study design

This study was conducted in a cross-sectional observational design using a questionnaire. The questionnaire collected sociodemographic characteristics of the students and incorporated two inventories to measure depressive and anxiety symptoms. The Beck Depression Inventory-II (BDI-II) was used to assess the presence of depressive symptoms [39]. The Beck Anxiety Inventory (BAI) was used to assess the presence of anxiety symptoms [40].

### Study population and sample size calculation

The sample size needed for this study was calculated using an online sample size calculator (http://www.raosoft.com/samplesize.html). The sample size was calculated based on a population of 2500 medical students using a 95% confidence interval (CI) and a 5% margin of error. The sample size was estimated based on arbitrary prevalence rate of depression and anxiety of 35% based on previous studies in the region [1–7, 16]. The sample size needed for this study was 307 medical students.

To achieve the sample size needed for this study, we decided to invite 425 medical students assuming a response rate of approximately 72% that was informed by our previous studies using a questionnaire as the study tool [41–50]. The target sample was stratified by academic year. Considering the number of students in each academic year, the target sample was stratified by academic year as 24% from the 1st year students, 20.9% from the 2nd year students, 16.2% from the 3rd year students, 16.9% from the 4th year students, 10.1% from the 5th year students, and 11.8% from the 6th year students.

Email was the main channel of communication between the medical students and the academic staff at the university where the study was conducted. A list of emails of all medical students enrolled in the MD program was obtained from the registrar office after permission was granted by the administration of the university. Contacts of strata were selected randomly and invitations were sent to potential participants via email. Those who did not respond receive 3 reminders, each was 1 week apart.

### The study tool

The study tool was a questionnaire that contained three sections. The first section collected sociodemographic characteristics of the students like age, gender, place where the student was born and raised before they were enrolled in the MD program, place of current residence while studying medicine, motives for studying medicine, if given a chance, would they study medicine again, academic stage (year of study), their Grade Point Average (GPA) out of 4, if they had ever failed a subject during their study of medicine, self-assessment of their mental health status (on a Likert-scale of 1–5, 1 = not at all mentally disturbed, 5 = having serious mental health problems), if they had ever attempted suicide, monthly household income, self-assessment of their financial status (on a Likert-scale of 1–5, 1 = completely dissatisfied with my financial status, 5 = completely satisfied with my financial status), marital status, self-assessment of their social life (on a Likert-scale of 1–5, 1 = completely dissatisfied with my social life, 5 = completely satisfied with my social life), and self-assessment of their religious commitment (on a Likert-scale of 1–5, 1 = completely dissatisfied with my religious commitment, 5 = completely satisfied with my religious commitment).

The second section contained the 21-item BDI-II. The 21 items of the BDI-II are shown in Additional file 1. The tool purported to screen for the presence of depressive symptoms. Participants could obtain a cumulative score of minimum of 0 to a maximum 63. Scores 0–13 indicated minimal depression, scores 14–19 indicated mild depression, scores 20–28 indicated moderate depression, and scores 29–63 indicated severe depression [39, 51]. The BDI-II was shown to have a sensitivity of 87% and a specificity of 79% [52].

The third section contained the 21-item BAI. The 21 items of the BAI are shown in Additional file 2. The tool purported to screen for the presence of anxiety symptoms. Again, the participant had to rate each item on a scale of 0–3. Scores 0–9 indicated normal or no anxiety, scores 10–18 indicated mild to moderate anxiety, scores 19–29 indicated moderate to severe anxiety, and scores 30–63 indicated severe anxiety [40, 53].

### Reliability

The questionnaire was in Arabic language. Validation of the Arabic versions of the BDI-II and BAI was reported previously [54–56]. The Arabic version of BDI-II and BAI were found to be internally consistent with a Cronbach’s alpha of 0.90 [54–56]. Prior to this study, a pilot was conducted with 29 medical students to assess the questionnaire for readability and comprehension [45, 46, 49]. The test re-rest reliability was excellent for both BDI-II and BAI as shown by Pearson’s r of 0.97 (95% CI 0.94 to 0.99) with a *p*-value < 0.001 for BDI-II and Pearson’s r of 0.96 (95% CI 0.91 to 0.98) with a *p*-value < 0.001 for BAI.

### Ethical approval

The study was conducted in accordance with the regulations and ethics followed at An-Najah National University and in compliance with the Declaration of Helsinki. The study received ethical approval from the Institutional Review Board (IRB) of An-Najah National University (Protocol # April-04-2018). Each participant provided written informed consent before taking part in the study.

### Statistical analysis

The data collected in this study were entered into IBM SPSS for Windows V.21.0 (IBM). The Kolmogorov-Smirnov test was used to assess if the data were normally distributed or not. As the data were not normally distributed, median, lower quartile (Q1) and upper quartile (Q3) with interquartile range (IQR) were used. Differences between scores were compared for statistical significance using Mann-Whitney *U* test or Kruskal Wallis tests. Correlation was investigated using Spearman’s correlation coefficients. We conducted a multiple linear regression analyses to identify the independent characteristics that were predictors of higher depression and anxiety scores. To control for confounders, characteristics that had a *p*-value < 0.05 in the Mann-Whitney *U* test, Kruskall-Wallis tests, and/or correlations were retained in the multiple linear regression. The goodness of fit of the regression was assessed using the adjusted R-squared with a *p*-value ≤0.05. Collinearity statistics were used to evaluate the variance inflation factor (VIF) and tolerance in the linear regression analyses. The VIF values were less than 1.87 and the tolerance values were greater than 1.04 indicated no multicollinearity problems with the independent variables [41, 45, 57, 58]. Statistical significance was considered when the *p*-value was ≤0.05.

## Results

### Response rate and characteristics of the participants

The questionnaire was distributed to 425 medical students. Valid and complete questionnaire were received from 286 participants giving a response rate of 67.3%.

The median age of the students who participated in the study was 20 with an IQR of 3 years and the median GPA was 3.2 with an IQR of 0.69. Of all students, 60.8% were female in gender, and 69.2% were born and raised in cities. However, 47.6% of the students were living in dormitories during their medical studies. When asked about their motives to study medicine, 81.8% of the students cited personal motives and 73.4% stated that they would study medicine if they were given a second chance. Of all students, 43.4% were in their clinical stage of medical studies (year 4 to year 6) and 74.5% of them stated that they had never failed a subject during their medical studies. In this study, 63.3% of the students had high monthly income, 3.1% were either married or engaged, and 4.5% stated that they had attempted suicide. When asked to self-rate their mental health status, financial status, social life, and religious commitment, 41.6, 56.6, 59.5, and 36.7% of the students rated these items 4 or 5 on the 5-point Likert-scale. The sociodemographic characteristics of the study participants are shown in Table 1.

**Table 1:** Characteristics of the study participants (*n = 286*)

Characteristic	n	%
**Age (years)**		
< 20	103	36.0
≥ 20	183	64.0
**Gender**		
Male	112	39.2
Female	174	60.8
**Place of residence (born and raised)**		
City	198	69.2
Village	82	28.7
Refugee camp	6	2.1
**Place of current residence during study of medicine**		
Family home	150	52.4
Dormitory	136	47.6
**Motives for studying medicine**		
Personal motives	234	81.8
Family motives	44	15.4
Undeclared	8	2.8
**If given a chance, would study medicine again?**		
No	76	26.6
Yes	210	73.4
**Academic stage**		
Basic (academic years 1–3)	162	56.6
Clinical (academic years 4–6)	124	43.4
**Grade Point Average (GPA) out of 4**		
< 3	89	31.1
> 3	197	68.9
**Ever failed a subject during your study of medicine**		
No	213	74.5
Yes	73	25.5
**Mental health status (self-assessed on a Likert-scale of 1–5)**		
1	19	6.6
2	59	20.6
3	89	31.1
4	94	32.9
5	25	8.7
**Ever attempted suicide**		
No	273	95.5
Yes	13	4.5
**Monthly household income (in US dollars)**		
Low (< 850)	15	5.2
Medium (850–1400)	90	31.5
High (> 1400)	181	63.3
**Financial status (self-assessed on a Likert-scale of 1–5)**		
1	4	1.4
2	24	8.4
3	96	33.6
4	105	36.7
5	57	19.9
**Marital status**		
Single	277	96.9
Engaged/Married	9	3.1
**Social life (self-assessed on a Likert-scale of 1–5)**		
1	9	3.1
2	33	11.5
3	74	25.9
4	96	33.6
5	74	25.9
**Religious commitment (self-assessed on a Likert-scale of 1–5)**		
1	26	9.1
2	41	14.3
3	114	39.9
4	96	33.6
5	9	3.1

### Prevalence of depressive and anxiety symptoms among medical students

When the BDI-II was self-administered, moderate depressive symptoms were prevalent in 14.0% of the students and 25.5% of the students had moderate to severe anxiety. Severe depressive symptoms were prevalent in 9.1% of the students and severe anxiety was prevalent in 21.3% of the students. Table 2 shows depressive and anxiety symptoms as indicated by BDI-II and BAI scores.

**Table 2:** Prevalence of depressive and anxiety symptoms among the study participants (*n = 286*)

Depression symptoms	n	%	Anxiety symptoms	n	%
Minimal depression (BDI-II scores 0–13)	162	56.6	Normal or no anxiety (BAI scores 0–9)	67	23.4
Mild depression (BDI-II scores 14–19)	58	20.3	Mild to moderate anxiety (BAI scores 10–18)	85	29.7
Moderate depression (BDI-II scores 20–28)	40	14.0	Moderate to severe anxiety (BAI scores 19–29)	73	25.5
Severe depression (BDI-II scores 29–63)	26	9.1	Severe anxiety (BAI scores 30–63)	61	21.3

In this study, moderate correlation was shown between scores of BDI-II and BAI (Spearman’s rho = 0.60, *p*-value < 0.001).

### Association between characteristics of the students with depressive symptoms

In this study, the median BDI-II score was significantly (*p*-value < 0.05) higher for students who cited family motives behind their choice to study medicine compared to those who cited personal motives or those who did not declare their motives. Similarly, the median BDI-II score was significantly (*p*-value < 0.05) higher for students who stated that they would not study medicine again if they were given a second chance compared to those who stated that they would. Students who were in the clinical stage (academic year 4 to academic year 6) had significantly (*p*-value < 0.01) lower BDI-II scores compared to students in the basic stage (academic year 1 to academic year 3). Students who had higher GPA and those who did not fail any subject during their medical studies had significantly (*p*-value < 0.05) lower BDI-II scores. Students who stated that they attempted suicide had significantly (*p*-value < 0.01) higher BDI-II scores than those who did not attempt suicide. Students who self-rated their mental health status, financial status, social life, and religious commitment as low had significantly (*p*-value < 0.001) higher BDI-II scores than those who self-rated these items higher.

Characteristics like age, gender, place where the student was born and raised, household income, and marital status were not significantly associated with the BDI-II scores (*p*-value > 0.05). Details of the associations between characteristics of the participants and symptoms of depression are shown in Table 3.

**Table 3:** Association between characteristics of the participants and symptoms of depression

Characteristic	n	%	Median	Q3	Q1	IQR	Mean rank	***p***-value	Correlation coefficient	***p***-value
**Age (years)**										
< 20	103	36.0	13.0	20.0	8.0	12.0	152.3	0.177	−0.02	0.699
≥ 20	183	64.0	11.0	19.0	6.0	13.0	138.6			
**Gender**										
Male	112	39.2	12.0	19.0	7.0	12.0	144.0	0.941	0.01	0.895
Female	174	60.8	12.0	19.0	6.0	13.0	143.2			
**Place of residence (born and raised)**										
City	198	69.2	12.0	19.0	7.0	12.0	145.4	0.613	0.01	0.897
Village	82	28.7	11.0	20.5	6.0	14.5	141.0			
Refugee camp	6	2.1	7.5	15.0	2.3	12.8	113.4			
**Place of current residence during study of medicine**										
Family home	150	52.4	11.0	18.0	6.0	12.0	134.7	0.059	0.11	0.054
Dormitory	136	47.6	13.5	20.0	8.0	12.0	153.2			
**Motives for studying medicine**										
Personal motives	234	81.8	11.0	18.0	6.0	12.0	137.9	0.048	0.14	0.015
Family motives	44	15.4	15.0	21.3	10.0	11.3	170.1			
Undeclared	8	2.8	12.5	19.5	11.8	7.8	162.1			
**If given a chance, would study medicine again?**										
No	76	26.6	13.5	21.0	8.0	13.0	160.4	0.037	−0.09	0.122
Yes	210	73.4	11.0	18.0	6.0	12.0	137.4			
**Academic stage**										
Basic (academic years 1–3)	162	56.6	13.0	21.0	8.0	13.0	155.8	0.004	−0.14	0.017
Clinical (academic years 4–6)	124	43.4	10.0	17.3	4.8	12.5	127.5			
**Grade Point Average (GPA) out of 4**										
< 3	89	31.1	15.0	25.0	8.0	17.0	160.4	0.020	−0.15	0.011
> 3	197	68.9	11.0	18.0	6.0	12.0	135.9			
**Ever failed a subject during your study of medicine**										
No	213	74.5	11.0	18.0	6.0	12.0	138.2	0.062	0.15	0.011
Yes	73	25.5	15.0	22.0	7.0	15.0	159.1			
**Mental health status (self-assessed on a Likert-scale of 1–5)**										
1 (least)	19	6.6	30.0	34.5	19.0	15.5	240.9	0.000	−0.55	0.000
2	59	20.6	19.0	26.0	15.0	11.0	206.2			
3	89	31.1	13.0	19.0	9.0	10.0	155.4			
4	94	32.9	7.0	11.8	4.0	7.8	93.4			
5 (most)	25	8.7	4.0	8.0	2.0	6.0	67.6			
**Ever attempted suicide**										
No	273	95.5	11.0	19.0	6.0	13.0	140.3	0.003	0.16	0.007
Yes	13	4.5	21.0	35.0	13.0	22.0	210.1			
**Monthly household income in US dollars**										
Low (< 850)	15	5.2	14.0	21.0	8.0	13.0	161.8	0.194	−0.1	0.091
Medium (850–1400)	90	31.5	13.0	20.8	7.3	13.5	153.8			
High (> 1400)	181	63.3	11.0	18.0	6.0	12.0	136.9			
**Financial status (self-assessed on a Likert-scale of 1–5)**										
1	4	1.4	9.5	20.3	8.0	12.3	151.5	0.003	−0.18	0.003
2	24	8.4	17.5	24.5	14.8	9.8	200.0			
3	96	33.6	13.0	21.0	7.0	14.0	149.6			
4	105	36.7	12.0	18.0	6.0	12.0	136.5			
5	57	19.9	9.0	16.0	4.0	12.0	121.9			
**Marital status**										
Single	277	96.9	12.0	19.0	7.0	12.0	143.2	0.762	0.02	0.762
Engaged/Married	9	3.1	15.0	21.0	6.0	15.0	151.7			
**Social life (self-assessed on a Likert-scale of 1–5)**										
1	9	3.1	20.0	24.0	18.0	6.0	214.1	0.000	−0.34	0.000
2	33	11.5	21.0	27.0	15.0	12.0	215.5			
3	74	25.9	13.0	20.8	6.0	14.8	149.6			
4	96	33.6	10.0	15.0	6.0	9.0	126.6			
5	74	25.9	9.0	16.0	4.0	12.0	118.6			
**Religious commitment (self-assessed on a Likert-scale of 1–5)**										
1	26	9.1	20.0	25.8	10.3	15.5	192.9	0.000	−0.29	0.000
2	41	14.3	17.0	24.0	11.0	13.0	181.5			
3	114	39.9	12.0	18.0	6.0	12.0	141.7			
4	96	33.6	10.0	15.0	4.0	11.0	120.1			
5	9	3.1	8.0	13.0	1.0	12.0	100.4			

*Q1* 1st quartile, *Q3* 3rd quartile, *IQR* interquartile range

We conducted a multiple linear regression analysis to control for confounding effects in a trial to explain the variance in BDI-II scores that were associated with some characteristics of the students who participated in this study. When characteristics that were shown to be significantly associated with BDI-II scores in the Mann-Whitney U, Kruskal-Wallis, and correlation tests were retained in the multiple linear regression analysis, characteristics like academic stage (*p*-value < 0.01), GPA (*p*-value < 0.01), mental health status (*p*-value < 0.001), ever attempted suicide (*p*-value < 0.05), and religious commitment (*p*-value < 0.01) remained significantly associated.

Other variables like motives for studying medicine, would study medicine again if given a second chance, self-rated financial status and social life were no longer significantly associated (*p*-value > 0.05). Details of the multiple linear analysis are shown in Table 4.

**Table 4:** Multiple linear regression analysis of association between characteristics of the participants and depression symptoms

Variable	Unstandardized Coefficients	S.E.	Standardized Coefficients	t	***p***-value
Motives for studying medicine	−0.12	0.82	−0.01	− 0.14	0.888
If given a chance, would study medicine again	0.20	1.13	0.01	0.18	0.856
Academic stage	−2.86	0.96	−0.14	−2.96	0.003
Grade Point Average (GPA)	−2.84	0.99	−0.13	−2.87	0.004
Mental health status	−4.54	0.47	−0.49	−9.58	0.000
Ever attempted suicide	5.12	2.16	0.11	2.37	0.019
Financial status	−0.42	0.51	−0.04	− 0.83	0.407
Social life	−0.73	0.49	−0.08	−1.50	0.135
Religious commitment	−1.46	0.49	−0.15	−2.99	0.003

### Association between characteristics of the students with anxiety symptoms

Anxiety symptoms as indicated by BAI scores were significantly higher (*p*-value < 0.05) in students who were less than 20 years old. Similar to BDI-II scores, BAI scores were significantly higher (*p*-value < 0.05) for students who cited family motives behind their choice to study medicine compared to those who cited personal motives or those who did not declare their motives. Again, BAI scores were significantly higher (*p*-value < 0.001) for students who were in their basic stage (academic year 1 to academic year 3) of medical studies compared to those in the clinical stage (academic year 4 to academic year 6). Students who stated that they had attempted suicide had significantly (*p*-value < 0.01) higher BAI scores than those who did not attempt suicide. Students with low income had significantly higher (*p*-value < 0.05) BAI scores compared to those with higher income. Students who self-rated their mental health status, financial status, social life, and religious commitment as low had significantly (*p*-value < 0.01) higher BAI scores than those who self-rated them higher.

Characteristics like gender, place where the student was born and raised, place of residence during medical studies, would study medicine again if given a second chance, GPA, ever failed a subject, and marital status were not significantly associated with the BAI scores (*p*-value > 0.05). Details of the associations between characteristics of the participants and symptoms of anxiety are shown in Table 5.

**Table 5:** Association between characteristics of the participants and symptoms of anxiety

Characteristic	n	%	Median	Q3	Q1	IQR	Mean rank	***p***-value	Correlation coefficient	***p***-value
**Age (years)**										
< 20	103	36.0	20.0	29.5	11.5	18.0	156.3	0.050	−0.11	0.066
≥ 20	183	64.0	16.0	26.0	9.0	17.0	136.3			
**Gender**										
Male	112	39.2	15.5	24.0	9.8	14.3	135.0	0.163	0.08	0.198
Female	174	60.8	19.0	28.0	11.0	17.0	149.0			
**Place of residence (born and raised)**										
City	198	69.2	18.0	27.0	12.0	15.0	145.6	0.434	−0.02	0.774
Village	82	28.7	18.0	27.0	8.0	19.0	136.0			
Refugee camp	6	2.1	26.0	34.8	13.5	21.3	175.0			
**Place of current residence during study of medicine**										
Family home	150	52.4	17.5	26.5	9.0	17.5	136.3	0.120	0.07	0.267
Dormitory	136	47.6	18.0	28.3	12.0	16.3	151.5			
**Motives for studying medicine**										
Personal motives	234	81.8	18.0	26.8	10.0	16.8	138.6	0.106	0.12	0.036
Family motives	44	15.4	22.0	29.3	11.8	17.5	166.3			
Undeclared	8	2.8	16.5	28.8	15.0	13.8	160.8			
**If given a chance, would study medicine again?**										
No	76	26.6	17.5	27.3	12.0	15.3	146.2	0.737	−0.01	0.916
Yes	210	73.4	18.0	27.0	10.0	17.0	142.5			
**Academic stage**										
Basic (academic years 1–3)	162	56.6	21.0	30.0	12.0	18.0	158.6	0.000	−0.19	0.001
Clinical (academic years 4–6)	124	43.4	15.0	22.0	8.8	13.3	123.8			
**Grade Point Average (GPA) out of 4**										
< 3	89	31.1	20.0	28.0	10.0	18.0	150.5	0.335	−0.05	0.379
> 3	197	68.9	18.0	27.0	10.0	17.0	140.3			
**Ever failed a subject during study of medicine**										
No	213	74.5	18.0	28.0	10.0	18.0	142.8	0.795	0.02	0.678
Yes	73	25.5	18.0	27.0	12.0	15.0	145.7			
**Mental health status (self-assessed on a Likert-scale of 1–5)**										
1	19	6.6	29.0	35.0	23.5	11.5	214.3	0.000	−0.48	0.000
2	59	20.6	24.0	33.0	16.0	17.0	188.6			
3	89	31.1	21.0	28.0	13.0	15.0	155.7			
4	94	32.9	12.0	19.0	8.0	11.0	106.6			
	25	8.7	6.0	16.0	4.0	12.0	78.5			
**Ever attempted suicide**										
No	273	95.5	18.0	27.0	10.0	17.0	140.7	0.009	0.15	0.014
Yes	13	4.5	27.0	31.0	22.0	9.0	201.6			
**Monthly household income in US dollars**										
Low (< 850)	15	5.2	28.0	35.0	20.5	14.5	194.8	0.044	−0.11	0.065
Medium (850–1400)	90	31.5	18.5	26.0	11.0	15.0	143.5			
High (> 1400)	181	63.3	17.0	27.0	10.0	17.0	139.3			
**Financial status (self-assessed on a Likert-scale of 1–5)**										
1	4	1.4	18.5	25.5	15.8	9.8	170.4	0.016	−0.19	0.001
2	24	8.4	27.5	31.5	17.5	14.0	192.0			
3	96	33.6	19.0	27.0	11.0	16.0	148.9			
4	105	36.7	16.0	25.0	9.0	16.0	134.6			
5	57	19.9	16.0	25.0	7.0	18.0	128.5			
**Marital status**										
Single	277	96.9	18.0	27.0	10.0	17.0	143.0	0.533	0.04	0.534
Engaged/Married	9	3.1	21.0	36.0	12.0	24.0	160.4			
**Social life (self-assessed on a Likert-scale of 1–5)**										
1	9	3.1	24.0	25.0	21.0	4.0	177.8	0.000	−0.25	0.000
2	33	11.5	27.0	35.0	16.0	19.0	194.3			
3	74	25.9	20.0	30.0	11.3	18.8	155.0			
4	96	33.6	16.0	24.0	9.0	15.0	131.5			
5	74	25.9	15.0	23.0	6.3	16.8	120.7			
**Religious commitment (self-assessed on a Likert-scale of 1–5)**										
1	26	9.1	18.5	30.0	14.3	15.8	167.0	0.143	−0.13	0.030
2	41	14.3	19.0	26.0	13.0	13.0	153.5			
3	114	39.9	18.0	28.0	11.0	17.0	147.9			
4	96	33.6	15.0	27.3	7.0	20.3	130.9			
5	9	3.1	13.0	22.0	3.0	19.0	108.7			

*Q1* 1st quartile, *Q3* 3rd quartile, *IQR*: interquartile range

Again, we conducted a multiple linear regression analysis to control for confounding effects in a trial to explain the variance in BAI scores that were associated with some characteristics of the students who participated in this study. When characteristics that were shown to be significantly associated with BAI scores in the Mann-Whitney U, Kruskal-Wallis, and correlation tests were retained in the multiple linear regression analysis, characteristics like academic stage (*p*-value < 0.05) and mental health status (*p*-value < 0.001) remained significantly associated.

Other variables like age, motives for studying medicine, ever attempted suicide, income, self-rated financial status, social life, and religious commitment were no longer significantly associated (*p*-value > 0.05). Details of the multiple linear analysis are shown in Table 6.

**Table 6:** Multiple linear regression analysis of association between characteristics of the participants and anxiety symptoms

Variable	Unstandardized Coefficients	S.E.	Standardized Coefficients	t	***p***-value
Age	−1.00	1.64	−0.04	−0.61	0.543
Motives for studying medicine	0.55	1.05	0.03	0.53	0.599
Academic stage	−3.42	1.58	−0.15	−2.16	0.031
Mental health status	−4.28	0.62	−0.40	−6.86	0.000
Ever attempted suicide	5.10	2.87	0.09	1.78	0.076
Monthly household income	0.29	1.21	0.02	0.24	0.810
Financial status	−0.61	0.79	−0.05	−0.77	0.441
Social life status	−0.79	0.64	−0.07	−1.23	0.221
Religious commitment status	−0.26	0.66	−0.02	−0.39	0.693

## Discussion

The present study is the first report on the prevalence of depressive and anxiety symptoms among Palestinian medical students and factors associated with symptoms of depression and anxiety. In this study, 23.1% of the medical students had moderate to severe depression and 46.8% had moderate to severe anxiety. High prevalence of depression and anxiety among medical students is a global phenomenon and despite the significant changes in medical education, this phenomenon continues to escalate on an apparently global level. While previous studies have investigated psychological distress among the general population or patients [59], little efforts were paid towards medical students in Palestine as an independent subgroup with distinct characteristics and predictors. The current study provides for the first time insights into the prevalence of depressive and anxiety symptoms among Palestinian medical students.

Prevalence rates of depressive and anxiety symptoms among Palestinian medical students were comparable to those reported in a meta-analysis of 62,728 medical students pooled from 77 studies which reported a prevalence rate of 28.0% [18]. Our findings showed that cumulatively, mild to severe depressive symptoms were prevalent in 43.4% and mild to severe anxiety symptoms were prevalent in 76.6% of the medical students who took part in the study. Previous studies have shown that prevalence rates of depression and anxiety varied significantly [8, 10, 11, 13, 16]. Depression was prevalent in 57.9, 40.4, 30.6, and 14.0% among medical students in Egypt [16], Bahrain [2], Estonia [12], and Lithuania [10], respectively. While anxiety was prevalent in 43.9, 51.0, 21.9, and 43.0% among medical students in Egypt [16], Bahrain [2], Estonia [12], and Lithuania [10], respectively. It has been argued that differences in prevalence rates could be explained by differences in culture and religion. Culture and religion have been shown to shape emotions.

It was not surprising that 4.5% of the students who participated in this study stated that they had attempted suicide. It has been argued that the psychological environments provided in medical schools are toxic where students are under constant academic and social pressures, demanding workloads, financial concerns, deprivation of sleep, mistreatments, and exposure to sufferings of the patients [1, 9, 60, 61]. These stressor factors affected medical students’ academic performance, dropout rates, substance abuse, and suicidal ideation [37, 62]. In this study, depressive and anxiety symptoms were significantly higher for students who cited family motives behind opting to study medicine. Probably, many medical students are persuaded and pressurized by their families to study medicine. Similarly, depressive symptoms were more severe in students who stated that would not consider studying medicine again if they were given a second chance. Depressive and anxiety symptoms were also significantly higher in students in their basic academic years (years 1–3) than those in clinical years (years 4–6). Our findings were consistent with those reported in previous studies [11, 17]. Depressive and anxiety symptoms were associated with the self-rated mental health, financial status, social life, and religious commitment. Findings of this study were consistent with those reported previously by Pillay et al. in which depressive and anxiety symptoms were significantly more in medical students with history of poor mental health [35]. The study also showed association between spirituality and depressive symptoms. Other studies that have examined financial status found that financial difficulties within the previous 12 months were considered to be a stressful life event [14]. A study conducted in Germany found an association between financial hardships with poor mental health [20]. Comparisons of depressive symptoms by gender among medical students yielded mixed findings with some showing no difference or a much higher prevalence among female medical students [4, 18]. In this study, gender was not associated with depressive and anxiety symptoms. Studies that found gender to be significant factor proposed that there were a multitude of reasons for female students to be depressed including social stigma, gender inequality, and cultural pressures [4, 18]. Our study found that the burden is shared equally between both genders. After controlling for confounding effects in the multiple regression analyses, academic stage and mental health status remained significantly associated with depressive and anxiety symptoms (Table 4 and Table 6). These results were consistent with those reported elsewhere [11, 17, 19, 35, 63].

In this study, good response rate (67.3%) was obtained and the number of students who completed the study was higher than some of the recent studies conducted in the region [2, 16]. The female gender was well represented (60.8%) in our sample, this representation reflected a similar percentage of female medical students in Palestine. Similarly, the vast majority (96.9%) of the students who took part in the study were single (Table 1). Little more than half (56.6%) of the sample was students in their basic stage (academic year 1 to academic year 3), again this mirrored approximately similar distribution of students in these stages.

Findings of this study provide insights into psychological distress and morbidity among medical students in Palestine. It has been argued that providing insights into psychological distress and morbidity might help set policies and plan cognitive, behavioral, and mindful interventions that might be effective in minimizing depressive and anxiety symptoms in these subpopulations [64, 65]. Our results could be helpful to policy and decision makers in healthcare and educational authorities to take measures and design interventions to reduce depressive and anxiety symptoms among medical students in Palestine.

### Limitations

Findings of this study be should interpreted considering the following limitations. First, this was a cross-sectional observational study. Ideally, our study should have been further explored in a longitudinal, large, prospective multicenter study with greater detail in examining stressors. Second, as the inventories in the current study were self-administered, it is likely that some students may have downplayed some of their depressive and anxiety symptoms or answered the question about previous suicidal attempts untruthfully due to fear of social stigma. Third, in the study, all students were recruited from one Palestinian university. However, to establish a true baseline for Palestinian medical schools, this research should have been conducted on a national level in all three Palestinian medical schools. It is noteworthy mentioning that the study was conducted in the largest university in Palestine. Fourth, although the sample size of this study was comparatively higher than those used in the studies conducted in the region [2, 16], a larger sample size could have increased the rigor of the current study. Fifth, although depressive and anxiety symptoms were assessed using two valid self-administered inventories, no further clinical assessments were conducted by psychologists or psychiatrists to assess depression and anxiety among the participants in this study. Sixth, the students who took part in this study were asked if they had ever attempted suicide. As the students could have depression and anxiety as a co-morbidity, answers of the students should be interpreted with caution as the findings could have been biased. Finally, we did not investigate the effects of family support, resilience, and coping skills of the students with regards to depression and anxiety. Many studies have shown that these factors could also improve health and impact prevalence of depression and anxiety among medical students [66, 67].

## Conclusions

Our findings strongly suggest that depressive and anxiety symptoms are prevalent among Palestinian medical students in a major university in the West Bank. Policy and decision makers in healthcare and educational authorities should consider addressing this issue of concern. Interventions designed to improve self-rated mental health, those targeting students in their early academic years, improving study conditions, and counseling to improve spirituality could be effective in reducing symptoms of depression among medical students in Palestine. However, interventions designed to improve self-rated mental health and those targeting students in their early academic years could be effective in reducing symptoms of anxiety among medical students in Palestine. Future studies are still needed to investigate if these interventions could be useful in reducing depressive and anxiety symptoms among Palestinian medical students.

## Supplementary information

**Additional file 1.** Items in the Beck Depression Inventory-II (BDI-II)

**Additional file 2.** Items in the Beck Anxiety Inventory (BAI)

## Data Availability

Data related to this study are either presented in the results section or in the additional files. Raw data can be obtained from the corresponding author upon reasonable request.

## Abbreviations

BAI: Beck Anxiety Inventory
BDI-II: Beck Depression Inventory-II
CI: Confidence interval
GPA: Grade Point Average
IQR: Interquartile range
Q1: Lower quartile
Q3: Upper quartile
VIF: Variance inflation factor
WHO: World Health Organization
