# Supplementary material for: Prevalence and factors associated with depressive and anxiety symptoms among Palestinian medical students
Source: BMC Psychiatry. 2020 May 19;20:244. doi: 10.1186/s12888-020-02658-1 (PMC7236464; doi:10.1186/s12888-020-02658-1)
Supplement: Supplementary file 1 — Additional file 1. Items in the Beck Depression Inventory-II (BDI-II) [file 12888_2020_2658_MOESM1_ESM.docx]

**Additional file 1:**

Items in the Beck Depression Inventory-II (BDI-II)

| **Item #** | **Item** |
| --- | --- |
| 1 | Sadness |
| 2 | Pessimism |
| 3 | Past failure |
| 4 | Anhedonia |
| 5 | Guilty feelings |
| 6 | Punishment feelings |
| 7 | Self-dislike |
| 8 | Self-criticalness |
| 9 | Suicidal thoughts |
| 10 | Crying |
| 11 | Agitations |
| 12 | Loss of interest |
| 13 | Indecisiveness |
| 14 | Worthlessness |
| 15 | Loss of energy |
| 16 | Change in sleep |
| 17 | Irritability |
| 18 | Change in appetite |
| 19 | Difficulty concentrating |
| 20 | Fatigue |
| 21 | Sexual interest |
