# Supplementary material for: Prevalence and factors associated with depressive and anxiety symptoms among Palestinian medical students
Source: BMC Psychiatry. 2020 May 19;20:244. doi: 10.1186/s12888-020-02658-1 (PMC7236464; doi:10.1186/s12888-020-02658-1)
Supplement: Supplementary file 2 — Additional file 2. Items in the Beck Anxiety Inventory (BAI) [file 12888_2020_2658_MOESM2_ESM.docx]

**Additional file 2**

Items in the Beck Anxiety Inventory (BAI)

| **Item #** | **Item** |
| --- | --- |
| 1 | Numbness or tingling |
| 2 | Feeling hot |
| 3 | Wobbliness in legs |
| 4 | Unable to relax |
| 5 | Fear of worst happening |
| 6 | Dizzy or lightheaded |
| 7 | Heart pounding/racing |
| 8 | Unsteady |
| 9 | Terrified or afraid |
| 10 | Nervous |
| 11 | Feeling of choking |
| 12 | Hands trembling |
| 13 | Shaky/unsteady |
| 14 | Fear of losing control |
| 15 | Difficulty in breathing |
| 16 | Fear of dying |
| 17 | Scared |
| 18 | Indigestion |
| 19 | Faint/lightheaded |
| 20 | Face flushed |
| 21 | Hot/cold sweats |
